# Variations in storm-induced bed level dynamics across intertidal flats

**DOI:** 10.1038/s41598-020-69444-7

**Published:** 2020-07-30

**Authors:** P. L. M. de Vet, B. C. van Prooijen, I. Colosimo, N. Steiner, T. Ysebaert, P. M. J. Herman, Z. B. Wang

**Affiliations:** 1 Hydraulic Engineering Department, Delft University of Technology, P.O. Box 5048, 2600 GA Delft, The Netherlands; 2 Department of Marine and Coastal Systems, Deltares, Delft, The Netherlands; 3 Department of Estuarine and Delta Systems, NIOZ Royal Institute for Sea Research, Utrecht University, Yerseke, The Netherlands; 4 Wageningen Marine Research, Wageningen University & Research, Wageningen, The Netherlands

**Keywords:** Geomorphology, Physical oceanography

## Abstract

Hydrodynamic forces on intertidal flats vary over a range of temporal and spatial scales. These spatiotemporal inhomogeneities have implications for intertidal flat morphodynamics and ecology. We determine whether storm events are capable of altering the long-term morphological evolution of intertidal flats, and unravel the contributions of tidal flow, wind-driven flow, waves, and water depth on inhomogeneities in bed level dynamics (bed level changes over ~days) across these areas. We complement decades of bed level measurements on eight intertidal flats in two estuaries in the Netherlands with an extensive 1-month field campaign on one of those flats. Across this intertidal flat, the hydrodynamics and morphodynamics of a storm event were captured, including the post-storm recovery. We show that individual events can persistently alter the morphological evolution of intertidal flats; magnitudes of some bed level changes are even comparable to years of continuous evolution. The morphological impacts of events are largely controlled by the relative timing of the forcing processes, and not solely by their magnitudes. Spatiotemporal variations in bed level dynamics of intertidal flats are driven by a combination of: (1) the inhomogeneous distributions of the hydrodynamic forcing processes (including the under-explored role of the wind); and (2) the linear proportionality between bed level dynamics and the local bed slope.

## Introduction

With accelerated sea level rise and anthropogenic interventions, increasing interest is being given by scientists and system managers to the long-term morphological evolution of estuarine intertidal flats^1–8^. These areas provide essential ecological values and ecosystem services^9,10^. Intertidal flats are also of interest for flood protection, as these areas serve as a buffer against storm waves^11,12^. The equilibrium state of intertidal flats has been thoroughly researched^13–15^, often considering a uniform bottom shear stress across these areas^16^. However, the existence of equilibria in the morphology of intertidal flats is questionable as the forcing processes change continuously over diverse time scales^13–15^. Intertidal flats evolve under spatiotemporally variable natural and anthropogenic forces^1–3,6–8^. In recent studies, (variations in) wind-driven flow^3–5^ and wind-driven waves^2^ were excluded in long-term model simulations on intertidal flat morphodynamics (i.e., assuming gradual changes over decades/centuries). In order to assess whether short-term fluctuations need to be included when studying long-term morphodynamics, it is key to understand the patterns in short-term bed level dynamics (defined as the bed level changes over $\sim$days, i.e., variations in the bed level evolution). Seasonal changes are relatively well described^17^, but the understanding of the role of storm events on the morphological evolution and bed level dynamics is still developing^18–21^. As bed level dynamics affect benthic macrofauna^22,23^ and salt marshes^24^, this understanding has also ecological relevance.

Previous studies emphasized the role of storm events on the sediment transport and morphology of intertidal flats^13,25–29^. The spatial distribution in hydrodynamic energy was used to explain erosion and accretion patterns following an individual event^29,30^. However, the individual contributions of the underlying hydrodynamic forces fluctuate within an event over various time scales^25,27,31–34^. Furthermore, the spatial distribution of hydrodynamic energy is site-dependent^25,29^. Therefore, for a more thorough understanding, the underlying variations in the hydrodynamic forcing need to be assessed. Only this knowledge can reveal if certain parts of intertidal flats are generally more vulnerable to events (i.e., are more dynamic) than other parts.

**Figure 1:**
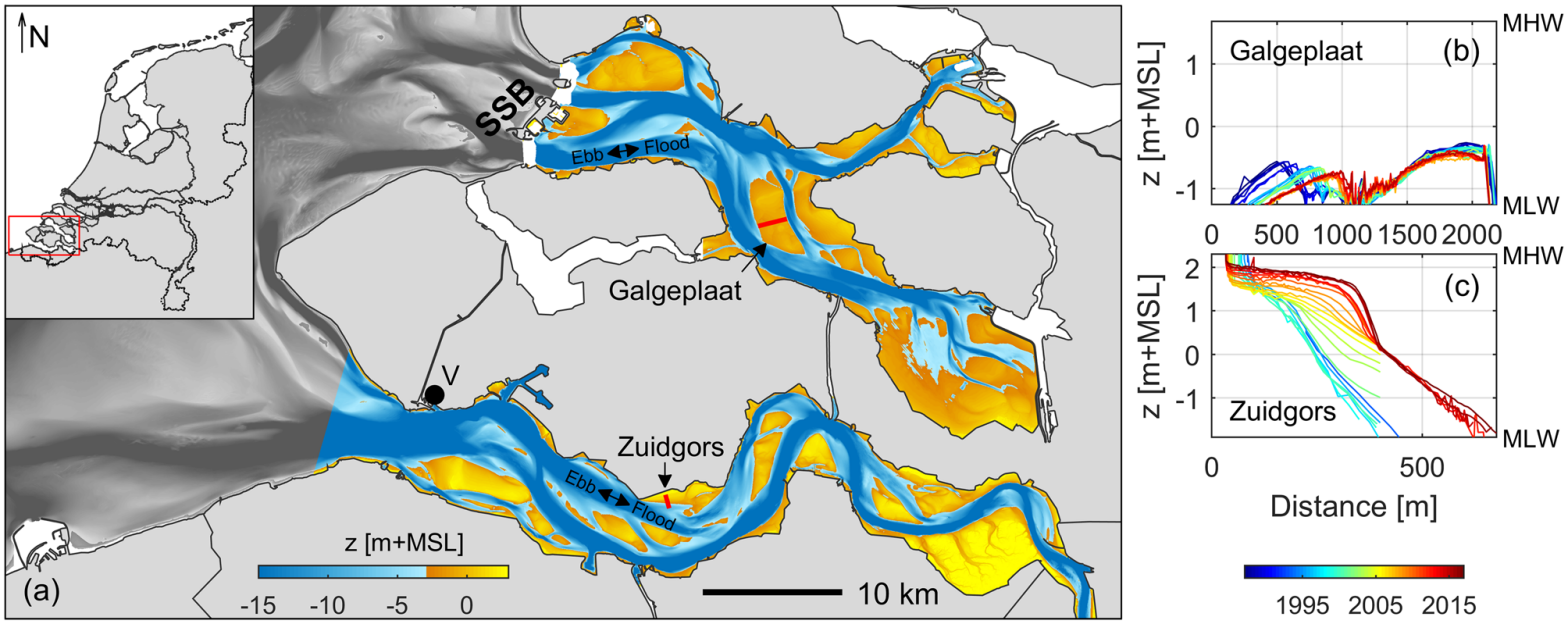
(**a**) Overview of the Eastern Scheldt (top estuary) with its storm surge barrier (SSB) and the Western Scheldt (bottom estuary), including their bathymetry. Both estuaries connect to the North Sea. The Zuidgors and Galgeplaat intertidal flats are indicated. The location of Vlissingen meteorological station (V) is indicated with the black marker. (**b**, **c**) The evolution of the red transects across the two intertidal flats over the past decades, vertically bounded between mean low water (MLW) and mean high water (MHW). All data is shown versus a fixed reference frame (the 2014 mean sea level; MSL).

This study aims at unravelling the effects of short-term events (on the scale of days) on the long-term morphodynamics (on the scale of years—decades) of intertidal flats. We consider eight intertidal flats in the Eastern Scheldt and Western Scheldt, the Netherlands (Fig. 1). These intertidal flats are gradually evolving (e.g., Fig. 1b, c) in response to human interventions (barriers and sediment relocations)^6–8^ and (related) large-scale changes in forcing processes^35–38^. We combine various datasets on these intertidal flats. With decades of high-resolution morphological measurements (average interval of 40 days; exceptionally small for decades of measurements), we identify the role of storm events on these evolutions and quantify the spatial inhomogeneities in bed level dynamics. Complementary measurements on the hydro-morphodynamics in the field provide insights on the forcing processes affecting the bed level dynamics. For 1 month we measured the hydro-morphodynamics over a cross-section on the Zuidgors intertidal flat (Western Scheldt; Fig. 1), capturing the bed level impact and recovery of a storm event (with 10-min averaged wind speeds of at most 22 m/s and above 15 m/s for 9 h). Furthermore, ecological implications were assessed by investigating the abundance of the benthic macrofauna before and after storm events. Finally, we explain the spatiotemporal patterns in bed level dynamics by considering both gradients in sediment transport and variations in bed slope across intertidal flats.

## Results

### Bed level dynamics on decadal time scales

**Figure 2:**
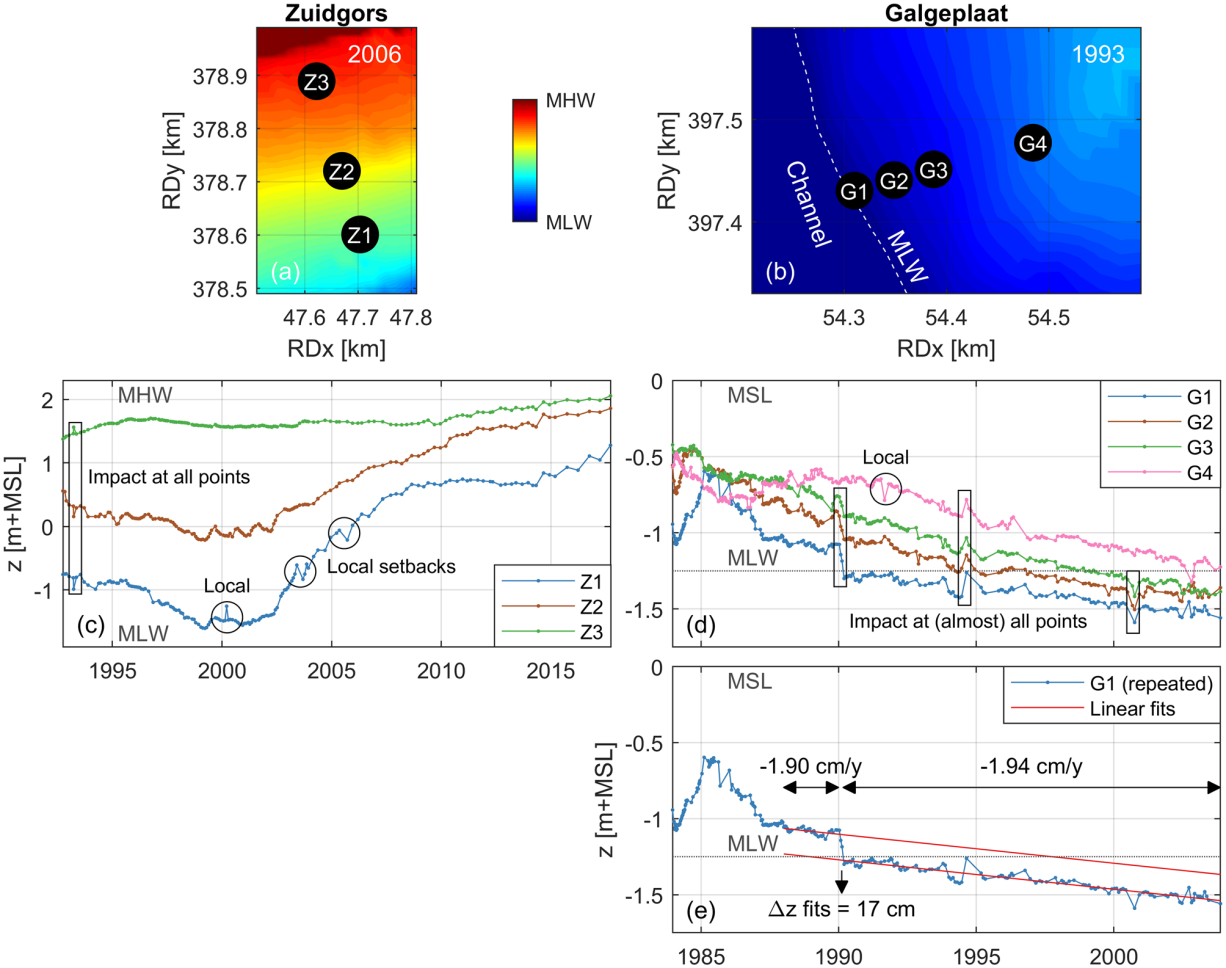
Bed level evolution of the point-elevation measurements. (**a**, **b**) Location of the sampling points Z1–Z3 on the Zuidgors intertidal flat (Western Scheldt) and G1–G4 on the Galgeplaat intertidal flat (Eastern Scheldt). See Fig. 1 for the position of these transects. The elevation maps, measured halfway the period of the time series, are shown as a reference. (**c**, **d**) Time series of the bed level evolution at Zuidgors and Galgeplaat, respectively. The black rectangular and circular annotations indicate examples of sudden changes in evolution. (**e**) The time series at Galgeplaat location G1 is repeated. Here, also a linear fit based on the data between 1988 and 1990 and a linear fit based on the data after February 1990 are shown (before and after the 1990 storm). All data is shown versus a fixed reference frame (the 2014 mean sea level; MSL).

Figure 2 presents the bed level evolution at fixed locations on two intertidal flats (Fig. 1a), measured on average 7 and 14 times a year, respectively. The Zuidgors intertidal flat (mean tidal range of 4.2 m; Western Scheldt) and Galgeplaat intertidal flat (mean tidal range of 2.9 m; Eastern Scheldt) are considered here. The measurement points (Z1–Z3; G1–G4) were 40 m to 175 m apart (Fig. 2a, b). Some short-term deviations along the long-term bed level trends, which were well in excess of the centimetre measurement precision, only affected specific locations (circles in Fig. 2c, d), while other impacts affected (almost) all measurement points (squares in Fig. 2c ,d). Even then, the impact was non-uniform (e.g., the first highlighted event in Fig. 2c). The intertidal flats recovered from various events in Fig. 2c, d within the next measurements, while other events affected the long-term evolution persistently.

Both identified setbacks (i.e., bed level changes against the long-term evolution) at Zuidgors (Fig. 2c) had a vertical impact similar to approximately 4 months of evolution (impact of $\sim$ 15 cm, evolution of $\sim$50 cm/year). The largest bed level change that persisted at Galgeplaat occurred in 1990 simultaneously at G1–G3 (Fig. 2d). The bed level changes resulted from a major storm event (26 February–2 March), characterized by 10-min averaged wind speeds that peaked locally at 27 m/s and were above 15 m/s for 73 h. The Eastern Scheldt has a storm surge barrier at its mouth (Fig. 1), that closed for four tides within this event, two more than any other storm in the record. This implied an absence of tidal forcing for a large part of the storm while wind-driven flow and waves affected the intertidal flat for long durations of near-constant water levels. Figure 2e shows the evolution of G1 including a linear fit based on the data measured during the two years before the storm, and a linear fit based on the data measured after the storm. The bed level evolution rate did not significantly change: − 1.90 cm/year before the storm and − 1.94 cm/year after the storm (both one order of magnitude larger than the sea level rise rate), implying no sign of recovery. The 17 cm of erosion during that event was equivalent to nine years of erosion at the average rate.

The bed level evolution at the highest location of Zuidgors Z3 (Fig. 2c) had a more gradual character than at the other two locations (Z1 and Z2). While these two lower sampling locations gained elevation in the long term, the variations in bed level (i.e., the bed level dynamics) reduced in time (i.e., their evolution became more gradual). This suggests that there may be a relation between the bed level dynamics and the bed elevation.

**Figure 3:**
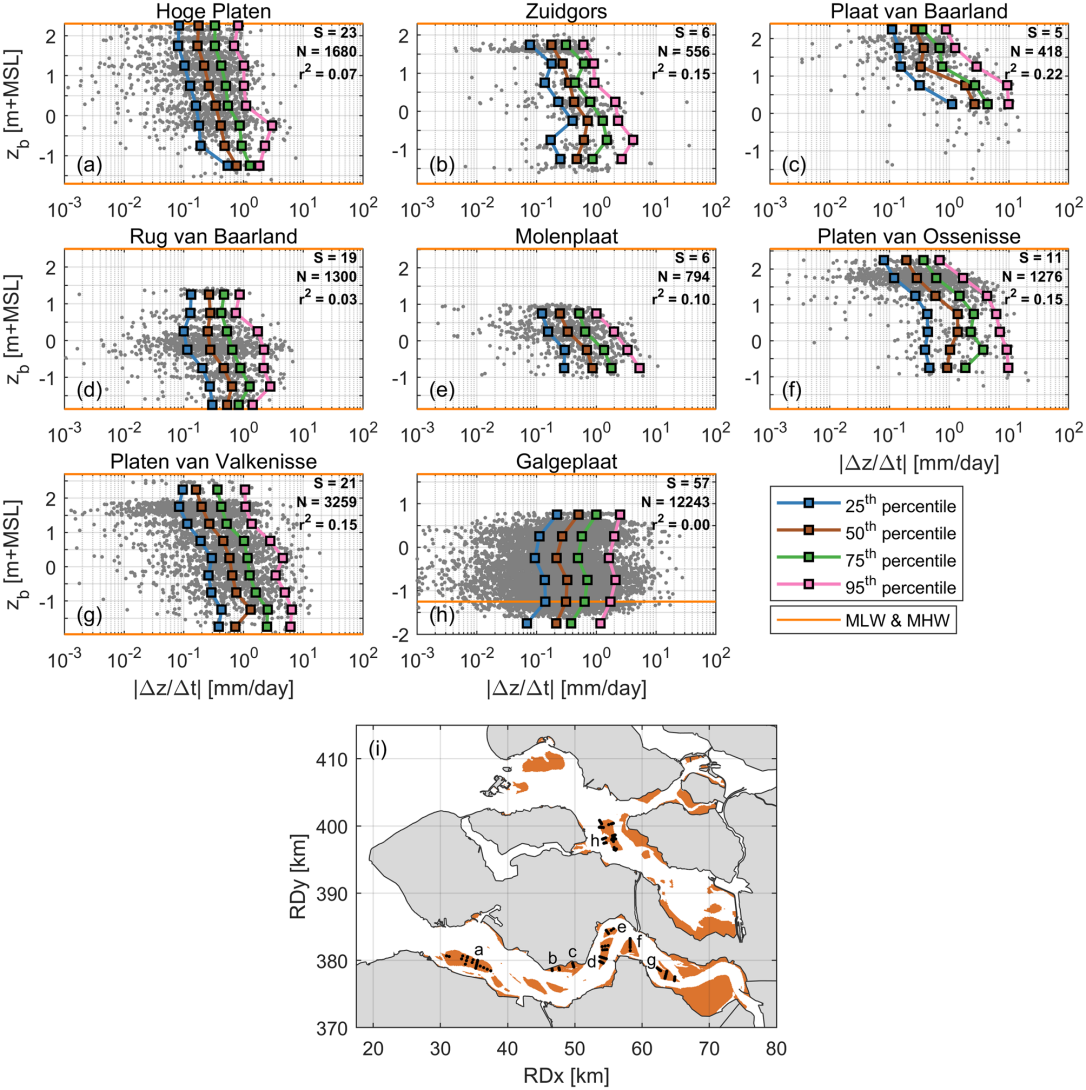
(**a**)–(**h**) Averaged bed level of two successive measurements versus the bed level rate of change between those measurements (considered as the bed level dynamics). The data is grouped per intertidal flat, see (**i**) for the location of intertidal flats a–h with the measurement locations indicated as black markers. The number of measurement locations for each flat (S), the total number of samples (N) and the coefficient of determination (${\hbox {r}}^2$) are shown in the top right corner for each flat. The horizontal grid lines represent the boundaries of the vertical bins over which the percentiles are computed. Percentiles are only shown for vertical bins that contained at least 20 data points. All plots are vertically bounded by mean low water (MLW) and mean high water (MHW), apart from (**h**) that contained also a substantial amount of data points below MLW. Absence of data at the top of the tidal window in (**d**), (**e**), and (**h**) is due to the limited height of these flats, while absence of data at the bottom of the tidal window is solely due to absence of measurements at those elevations. All data is shown versus a fixed reference frame (the 2014 mean sea level; MSL).

With Fig. 3, the existence of such a relation is tested for intertidal flats in the Eastern Scheldt and Western Scheldt. Only locations with sufficient data are considered (Fig. 3i): seven intertidal flats in the Western Scheldt and only the Galgeplaat in the Eastern Scheldt. The bed level dynamics are computed by dividing the absolute elevation change of two successive measurements (after removal of the 5 year trend) by the time interval. The bed level dynamics are shown versus the average elevation of the two successive measurements. For each intertidal flat, data from all measurement locations were merged. The average duration between successive measurements was 40 days. For each bin of 0.5 m, the 25th, 50th, 75th, and 95th percentiles were determined.

The vertical trends are relatively consistent along the different percentiles. Consistently for all sites in the Western Scheldt (i.e., excluding Galgeplaat), the bed level dynamics reduce by approximately one order of magnitude from the lower to the higher part of the flat. In contrast, such a pattern in bed level dynamics was not observed for the Galgeplaat intertidal flat, where the bed was actually more dynamic above MSL (mean sea level). In the Western Scheldt 3–22% of the variance in the logarithm of the bed level dynamics is explained by elevation alone, whereas none of this variance is explained by elevation for the Eastern Scheldt samples (see coefficients of determination in Fig. 3). Therefore, there is a relation between the bed level dynamics and bed elevation in the Western Scheldt. However, as bed elevation alone explains only part of the variance, the bed level dynamics are also affected by other aspects. The role of the hydrodynamic forcing processes is studied in the next sections.

### Inhomogeneous morphological impact of a storm event

**Figure 4:**
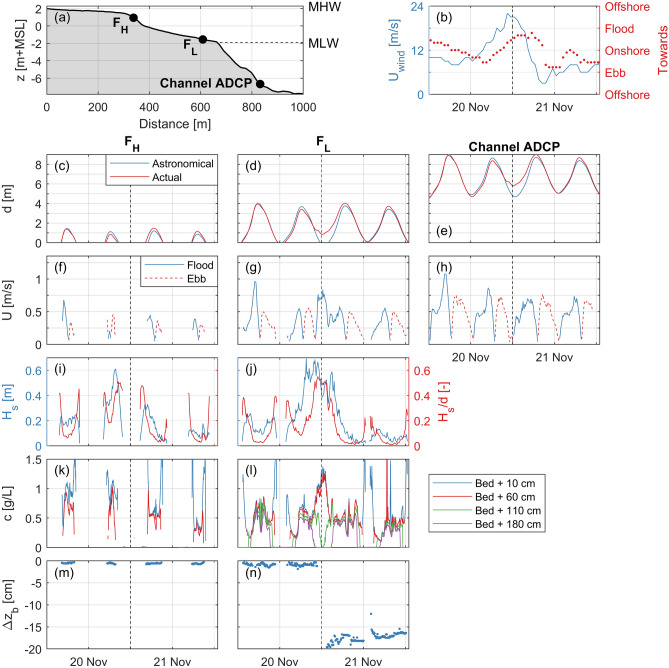
Process measurements for four tides along a storm event in 2016 on the Zuidgors intertidal flat (Western Scheldt). (**a**) Measurement stations (see Fig. 1a for the location of the transect). (**b**) Hourly-averaged wind speeds and directions at Vlissingen meteorological station (location in Fig. 1). (**c**)–(**e**) Water depths, including the derived astronomical water depths (the difference is the storm surge). (**f**)–(**h**) Flow velocity magnitudes (flood and ebb directions indicated in Fig. 1). (**i**, **j**) Significant wave heights (the wave logger of ${\hbox {F}}_{\mathrm{H}}$ was positioned 50 m downslope), including the significant wave height over depth ratio. (**k**, **l**) Suspended sediment concentrations at various distances from the bed. (**m**, **n**) Bed level changes relative to the initial elevations. The vertical black dashed lines indicate the peak of the storm. All time series are in CET (UTC+1).

The long-term data infer a spatiotemporally inhomogeneous character in the bed level dynamics of intertidal flats. To reveal the hydrodynamic mechanisms that drive this spatiotemporal inhomogeneity, measurements of wind, water levels, flow, waves, suspended sediment concentrations, and bed levels during a single storm event (20 November 2016) were analysed in detail. Time series of these processes at three stations at the transect across the Zuidgors intertidal flat (Fig. 1a) are shown in Fig. 4b–n. As visualised in Fig. 4a, these three stations consist of an ADCP in the channel, the low elevated frame ${\hbox {F}}_{\mathrm{L}}$ at 0.4 m above MLW (mean low water), and the high elevated frame ${\hbox {F}}_{\mathrm{H}}$ at 1.0 m above MSL.

The peak of the storm (hourly-averaged wind speeds up to 22 m/s, Fig. 4b) coincided with low water (Fig. 4c–e). The storm surge reached 1.3 m on top of the astronomical water level at Zuidgors. ${\hbox {F}}_{\mathrm{L}}$ was submerged during the storm peak (Fig. 4d), while it would have been emerged in absence of the storm. The water depth at this station was 0.8–1.3 m for four successive hours around low water. By contrast, ${\hbox {F}}_{\mathrm{H}}$ was emerged during the peak of the storm.

Under mild wind conditions (e.g., the first and last tide in Fig. 4), the velocities were flood-dominant, with maximum velocities occurring just before high water. The velocities were predominantly alongshore directed (not shown here), with spring-tidal velocities near the MLW line up to 1.5 m/s. The velocity magnitudes gradually decreased towards higher bed elevations of the flat.

The wind affected the flow on the flat substantially. While the flow in the channel reversed precisely at low water (Fig. 4h), the flow at ${\hbox {F}}_{\mathrm{L}}$ reversed 2 h before (Fig. 4g), even though the two locations were only 200 m apart. At ${\hbox {F}}_{\mathrm{L}}$, the velocity exceeded 0.7 m/s for 2 h around low water, while the velocity around low water is normally one order of magnitude smaller or even absent (by emergence of the bed). The flow at ${\hbox {F}}_{\mathrm{H}}$ was also modified: it was almost solely ebb-directed in the tide preceding the storm peak (second tide in Fig. 4f). The wind was in ebb direction during the largest part of the tide preceding the storm peak, only at the end of this tide preceding the storm peak (when ${\hbox {F}}_{\mathrm{H}}$ was emerged) the wind was in flood direction (Fig. 4b). The wind remained in flood direction for 9 h in the tide following the storm peak. The largest amplification of the flow occurred at ${\hbox {F}}_{\mathrm{L}}$ (in flood direction), as ${\hbox {F}}_{\mathrm{H}}$ was emerged during the peak of the storm. But even with the 0.7 m/s amplification at ${\hbox {F}}_{\mathrm{L}}$ during low water, larger velocities occurred during calmer wind conditions (e.g., first tide in Fig. 4f). However, these larger velocities occurred for larger water depths and for shorter durations.

Waves on the flat were limited by the water depth for a large part of the storm. Significant wave heights exceeded 0.60 m at both frames. However, waves at ${\hbox {F}}_{\mathrm{H}}$ were for a large portion of time absent (by emergence of the bed) or depth-limited (at most 50% of the water depth at this intertidal flat). Even though ${\hbox {F}}_{\mathrm{L}}$ was submerged during the peak of the storm, waves were still depth-limited for more than three successive hours around low water.

Suspended sediment concentrations were largely affected by the storm. Suspended sediment concentrations were measured at 0.1 m and 0.6 m from the bed at both frames, and also at 1.1 m and 1.8 m from the bed at ${\hbox {F}}_{\mathrm{L}}$ (Fig. 4k–l). The concentrations were relatively uniform over the water column (differences smaller than 30%). Concentration peaks are observed during flow peaks and during conditions for which the wave heights were depth-limited. These two conditions did not occur simultaneously for the tides with mild wind conditions. However, during the low water at the storm peak, the amplified flow and breaking waves coincided for hours at ${\hbox {F}}_{\mathrm{L}}$. As a result, concentrations exceeded 1 g/L for 2.5 h. This concentration peak lasted one order of magnitude longer than the other observed concentration peaks.

The morphological changes by the storm event varied across the flat. The changes in bed level at ${\hbox {F}}_{\mathrm{H}}$ were less than half a centimetre during these four tides. The storm did not cause a distinctive impact here (Fig. 4m). Conversely, the bed level at ${\hbox {F}}_{\mathrm{L}}$ lowered 20 cm during the storm (Fig. 4n). This drop in elevation occurred within a 3 h window around low water at the peak of the storm. This 3 h window at ${\hbox {F}}_{\mathrm{L}}$ coincided precisely with the windows during which the water depth was limited to 0.8–1.3 m, the velocity was amplified to $\sim$ 0.7 m/s, the waves experienced depth-induced breaking, and the suspended sediment concentrations were above 1 g/L. A comparable simultaneous peaking of the forcing processes for hours did not occur at ${\hbox {F}}_{\mathrm{H}}$.

### Identifying patterns in forcing processes

**Figure 5:**
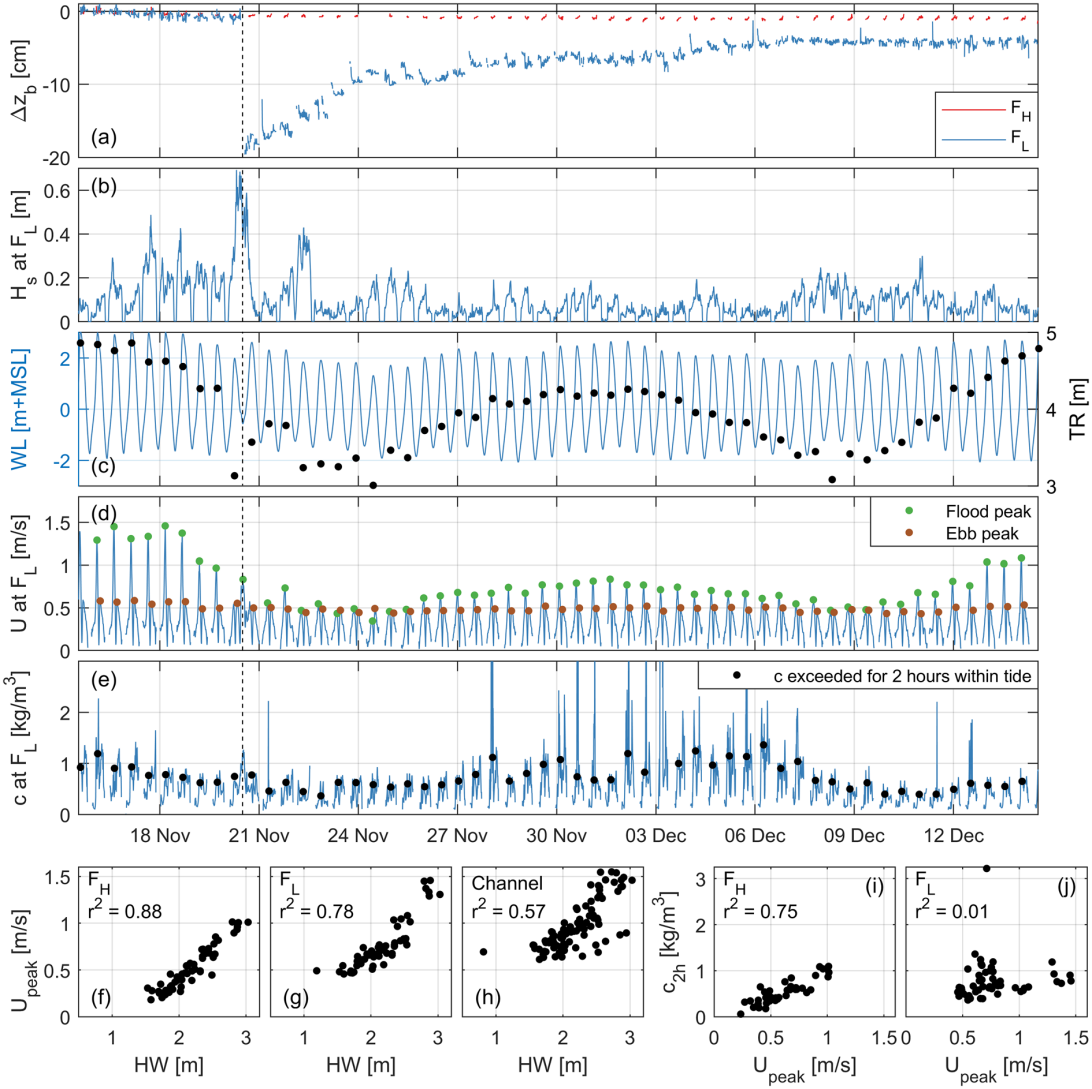
Process measurements for the full 1 month campaign on the Zuidgors intertidal flat (Western Scheldt; see Figs. 1a and 4a for the locations). (**a**) Bed level changes for both frames relative to the initial elevations. (**b**) Significant wave heights at ${\hbox {F}}_{\mathrm{L}}$ (near mean low water). (**c**) Water levels as measured in the channel (indicative for the full flat), the markers indicate the tidal range. (**d**) Flow velocity magnitudes at ${\hbox {F}}_{\mathrm{L}}$, the markers indicate the flood and ebb peaks. (**e**) Suspended sediment concentrations 60 cm above the bed at ${\hbox {F}}_{\mathrm{L}}$, the markers indicate the concentration that was exceeded for 2 h. (**f**)–(**h**) the maximum velocity of each tide versus the high water level. (**i**, **j**) the suspended sediment concentrations that were exceeded for 2 h versus the maximum velocity of each tide. The vertical black dashed lines indicate the peak of the storm of Fig. 4. All time series are in CET (UTC+1).

**Figure 6:**
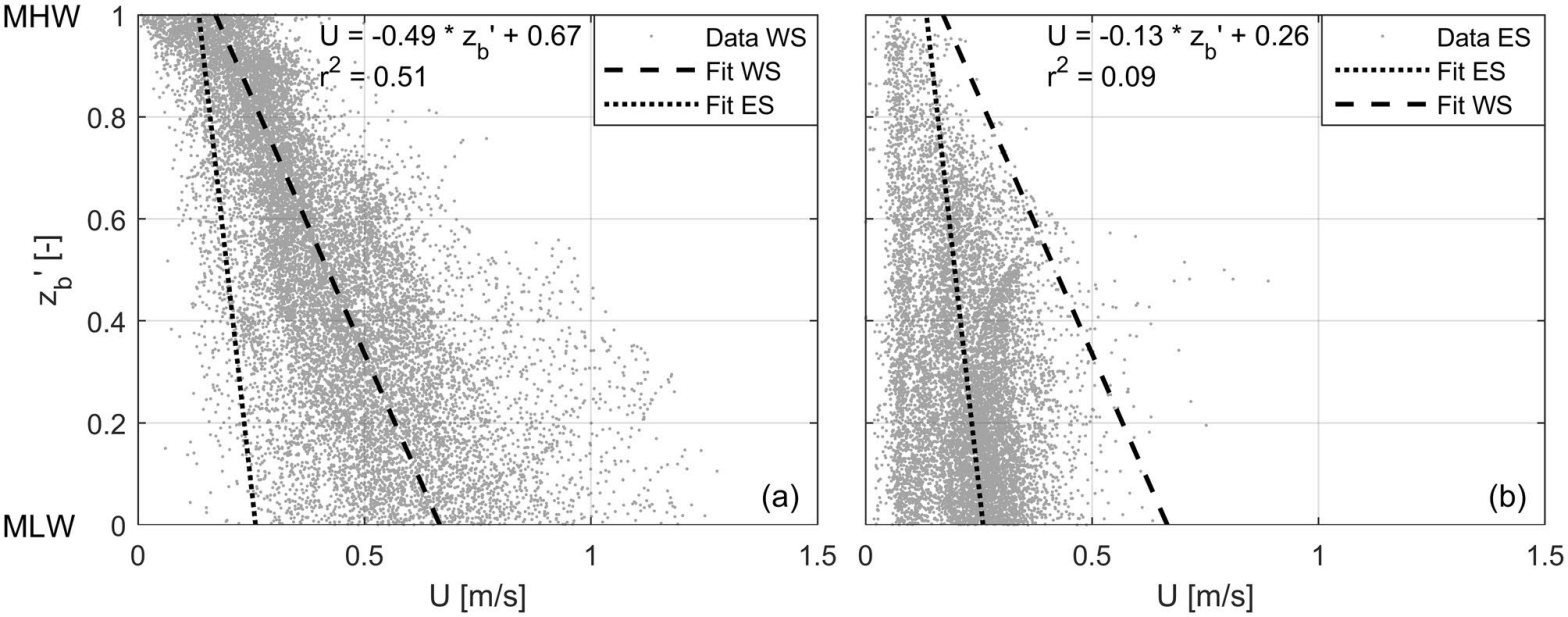
Bed level, scaled along mean low water (MLW; 0) and mean high water (MHW; 1), versus the average peak flow velocity from a 1 month simulation. All points on the intertidal areas of the full (**a**) Western Scheldt (WS) and (**b**) Eastern Scheldt (ES) are shown. Linear fits are provided with coefficients of determination (${\hbox {r}}^2$). The fits of both estuaries are shown in both graphs to allow comparisons.

**Figure 7:**
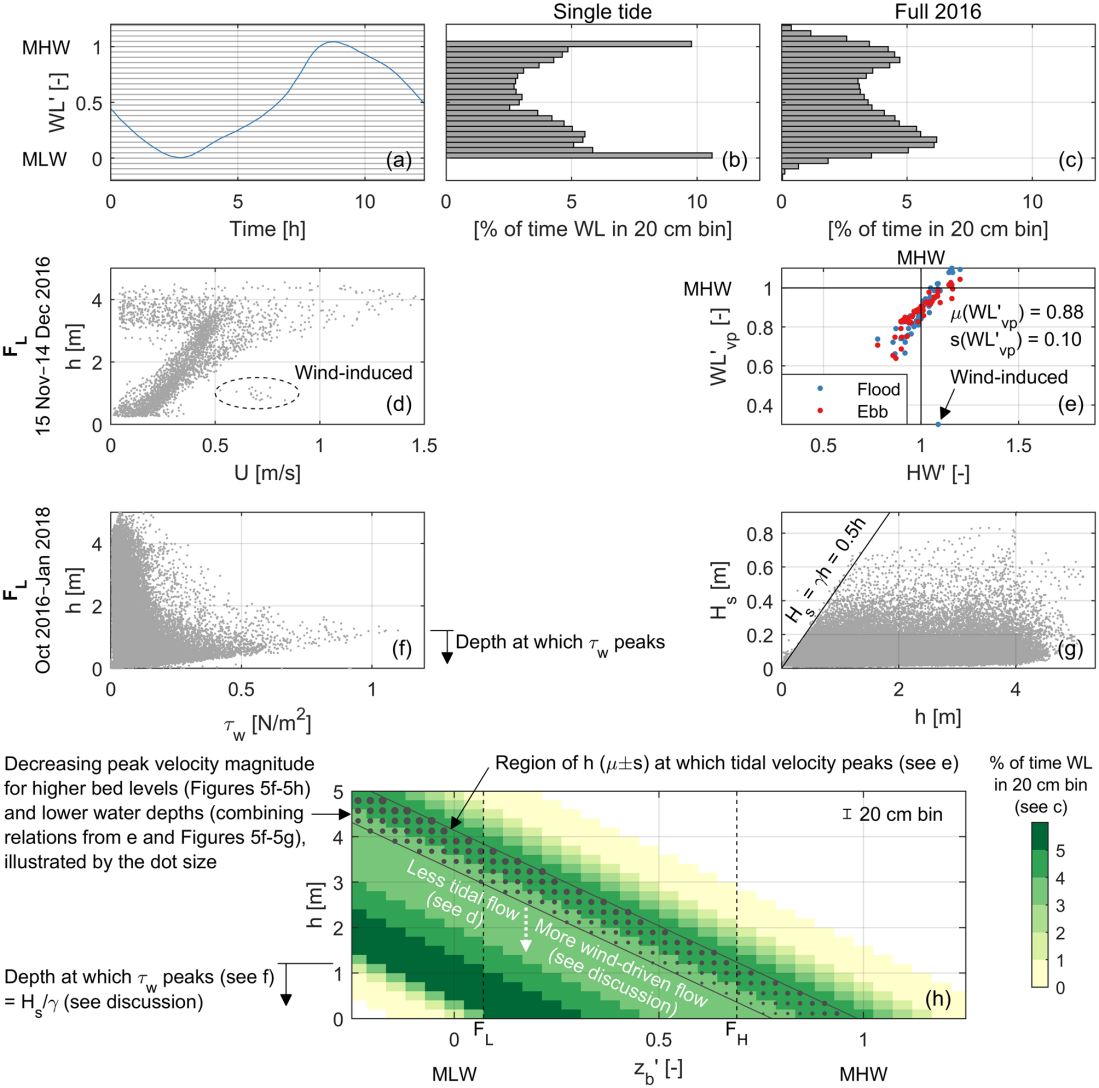
Distributions in hydrodynamic forcing for variations in water depth and bed level. (**a**) Water level time series over a single tidal cycle (29 November 2016) at the Zuidgors intertidal flat (Western Scheldt). The horizontal lines indicate 20 cm bins. (**b**) The corresponding probability distribution of the water level across these bins. (**c**) The probability distribution of the water level over a full year (2016). (**d**) Water depth versus flow velocities at ${\hbox {F}}_{\mathrm{L}}$ (10 min interval). The wind-induced flow during the storm event of Fig. 4 is marked with the ellipse. (**e**) Water levels at which the velocities peak at ${\hbox {F}}_{\mathrm{L}}$, versus the high water of each tide. The storm of Fig. 4 is indicated. Both axes are scaled along mean low water (MLW; 0) and mean high water (MHW; 1). (**f**) Water depth versus the wave-induced shear stresses at ${\hbox {F}}_{\mathrm{L}}$ (10 min interval). (**g**) Significant wave height versus water depth measured at ${\hbox {F}}_{\mathrm{L}}$ (10 min interval), with the wave-breaking index $\gamma$ indicated. (**h**) For a range in bed levels (scaled along MLW and MHW) the probability that the water depth is within a certain 20 cm bin is indicated with the colours, using the distribution of (**c**). The diagonal dotted region indicates the water depths at which the tidal velocities peak, using the average and standard deviation of (**e**). The range of depths at which the wave-induced shear stress peaks, based on (**f**), are indicated on the left. The vertical dashed lines show the position of the frames.

The spatiotemporal variability in the forcing processes drove the inhomogeneity of the storm impact. In this section, we aim to unravel the long-term patterns of the hydrodynamic forcing processes across the Zuidgors tidal flat, which is relevant to understand the related bed level dynamics patterns. For this aim, the processes measured in the full 1-month measurement campaign at Zuidgors are analysed (Fig. 5) and relations between the processes are derived. A relation between the flow velocity and the bed elevation is not only assessed for this specific tidal flat, but is also tested for all intertidal areas in the Eastern Scheldt and Western Scheldt (Fig. 6). Finally, the distributions, relative timing, and importance of the various hydrodynamic processes across the intertidal flat are unravelled by integrally assessing the identified relations between the processes (Fig. 7).

The 1-month measurement campaign at Zuidgors shows also in the calm periods a substantial spatiotemporal variability in the bed level changes (Fig. 5; the vertical dashed lines highlight the storm discussed in Sect. 2.2). Significant wave heights were, apart from the highlighted storm event, below $\sim$ 0.4 m and for most tides even below $\sim$ 0.2 m. Figure 5a indicates that the bed level at ${\hbox {F}}_{\mathrm{H}}$ was not only relatively stable during the storm, but also over the full month (less than 2 cm erosion over 30 days). Before the 20 cm erosion event, the bed level at ${\hbox {F}}_{\mathrm{L}}$ was relatively stable (comparable evolution as at ${\hbox {F}}_{\mathrm{H}}$). In the three days after the storm, half of the erosion (0.1 m) recovered. After two weeks, the bed returned to a relatively stable and similar evolution as at ${\hbox {F}}_{\mathrm{H}}$. However, 25% of the erosion (5 cm) persisted.

In the 1-month campaign, the tidal forcing was almost the smallest for the tides in the highlighted storm event (Fig. 5c; almost the smallest tidal range). The tidal range fluctuated over the month between 3.0 and 4.9 m. These fluctuations resulted mainly from spring-neap fluctuations (spring tide at the beginning, halfway, and end of the record), which were affected by storm surges (up to 1.3 m for the storm indicated with the vertical dashed lines in Figs. 4e and 5c). Spring-neap fluctuations were reflected in the peak flood velocities, with variations between 0.35 and 1.46 m/s (a ratio of maximum variation of 4.2) at ${\hbox {F}}_{\mathrm{L}}$ (Fig. 5d). Fig. 5f–h show a proportionality between the high water level and the peak velocity for all measurement locations. On the flat, 78–88% of the variance in the peak velocities was explained by the high water level, in the channel this was 57%. The tidal range is a similar indicator for the peak velocities which explains 77–83% of the variance on the flat and 81% in the channel (not shown in these figures). The spring-neap fluctuations in the ebb peaks were much less, these varied only between 0.43 and 0.58 m/s (a ratio of maximum variation of 1.3; Fig. 5d). Nevertheless, the consequential variation in velocity asymmetry (relative magnitude of flood versus ebb velocities) across the spring-neap cycles did not affect the relation between peak velocity and high water level, as almost all tides were flood-dominant.

The suspended sediment concentrations varied with the spring-neap cycles (Fig. 5e; measured 0.6 m above the bed). This is especially visible in the values that were exceeded for 2 h each tide (less sensitive to outliers than the maxima). However, even though the spring tides in the middle of the record did not feature the largest tidal range (nor high water level) and peak velocities in the record, the concentrations were higher than during the preceding and following spring periods. This is expressed in Fig. 5j as well, which shows no clear relation (${\hbox {r}}^2$ of 0.01) between the concentrations that were exceeded for 2 h and the peak velocities at ${\hbox {F}}_{\mathrm{L}}$. However, these concentrations did correlate well to the peak velocities at ${\hbox {F}}_{\mathrm{H}}$ (${\hbox {r}}^2$ of 0.75).

Not only at Zuidgors do the peak flow velocities generally decrease for increasing bed elevations (Fig. 5f–h). For the full Western Scheldt and Eastern Scheldt (which include also tidal flats surrounded by channels) are the peak velocities generally smaller at higher bed elevations (Fig. 6; modelled average peak velocities). In the Western Scheldt 51% of the variance in the average peak velocities on the intertidal flats is explained by only the elevation, in the Eastern Scheldt (which has closed branches) this is 9%. Almost all data points in the Eastern Scheldt are below the linear fit of the Western Scheldt. There is thus an inhomogeneity in flow velocities over different elevations, within an estuary (spread across the linear fits), and between different estuaries.

Also over long time scales, the distributions of both the occurrence and strength of the forcing processes vary across intertidal flats. The remainder of this section focusses on unravelling the relative importance of the tide, waves, and wind over different water depths and bed elevations at the Zuidgors intertidal flat (Fig. 7). The result is implemented in a schematic diagram, with the importance of the processes visualized over both bed elevation and water depth (Fig. 7h). The background colour represents the probability of occurrence of a water depth at a certain bed elevation (i.e., the relative duration). This probability of occurrence of a water depth results directly (water level–bed level) from the water level distribution (Fig. 7a, b). Here, the distribution over a full year is considered, to include surge and spring-neap fluctuations (Fig. 7c). Even with these fluctuations, the probability of occurrence of a water level near MLW and MHW (mean high water) is the largest. There is hence a variation in probability of occurrence of a certain water depth across the intertidal flat (Fig. 7h).

Tidal flow velocities generally decrease with water depth (Fig. 7d). Outliers are part of the wind event of Fig. 4, illustrating the effect of the wind. At large water depths, smaller velocities occur because of the reversal of the tidal flow. The average water level at which the tidal flow velocities peak equals $0.88{\pm } 0.10$ (scaled between MLW and MHW) at ${\hbox {F}}_{\mathrm{L}}$ and almost identically $0.93 {\pm } 0.10$ at ${\hbox {F}}_{\mathrm{H}}$ (Fig. 7e). Therefore, the water depth at which tidal velocities peak decreases almost linearly with an increase in bed elevation (the dotted region in Fig. 7h), which goes together with a decrease in peak velocity magnitude (Figs. 5f–h, 6). Furthermore, the higher the water level at which tidal flow velocities peak (i.e., larger water depths), the larger the magnitude of these peak flow velocities. This is a consequence of (1) an increase of the water level at which the tidal flow velocity peaks for an increase of the high water level of the tide (${\hbox {r}}^2 = 0.85$ at ${\hbox {F}}_{\mathrm{L}}$; Fig. 7e) and (2) larger peak flow velocities for higher high water levels (especially on the flat, Fig. 5f–g). Peak flow velocities decrease hence both for higher bed elevations and smaller water depths (illustrated by the dot size in Fig. 7h).

Waves are limited by the water depth. At this location, the significant wave height does not exceed half the water depth (Fig. 7g), with the same wave-breaking index at ${\hbox {F}}_{\mathrm{H}}$ (not shown in the figure). Within the 16 months of wave measurements, the maximum wave-induced shear stress occurred for water depths smaller than 1.2 m (Fig. 7f), which is roughly twice the maximum observed significant wave height (Fig. 5b). These limited water depths for which waves are most important occur the longest at the lowest part of the intertidal flat (Fig. 7h).

### Inhomogeneous impact on benthic macrofauna

**Figure 8:**
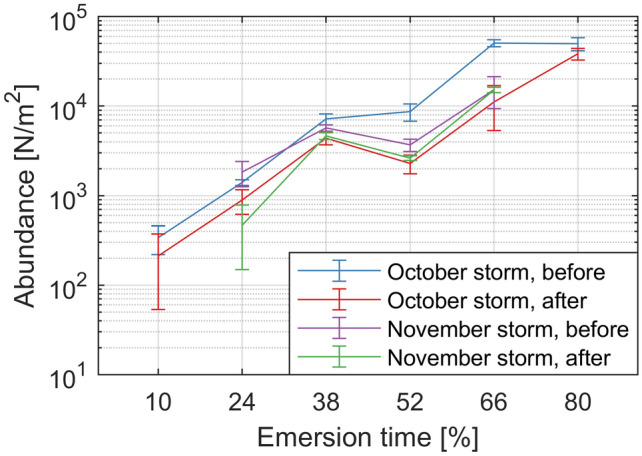
The abundance of benthic macrofauna (number of individuals per ${\hbox {m}}^2$) across six stations equally spaced in emersion time on the Zuidgors intertidal flat (Western Scheldt). Benthic macrofauna were sampled around the 5 October 2017 and the 23 November 2017 storm events, with at most 10 days between the before storm and the after storm measurements. The error bars (one standard deviation above and below) are based on three replicates.

As bed level dynamics may affect benthic macrofauna (and the other way around), we investigate whether the spatiotemporal inhomogeneity of storm impacts has consequences for the benthic macrofauna on the Zuidgors intertidal flat. Figure 8 presents the logarithm of the abundance (mean and standard deviation) of benthic macrofauna before and after two storms in 2017 (in October and November, respectively; i.e., different storms than the one of Fig. 4) over six stations along a cross-shore transect of 210 m on the Zuidgors intertidal flat (1 km west of the frames of Fig. 4). During both storm events, the hourly-averaged wind speed exceeded 15 m/s for several hours.

In general, the abundance increased along the cross-shore transect with a higher emersion time (i.e., higher bed elevation). Storm events imposed substantial reductions in abundance. These changes in abundance occurred spatially inhomogeneous, just as observed for the morphological changes (e.g., Fig. 4m–n). For example, the highest two stations (66% and 80% emersion time) had an almost equal quantity of benthic macrofauna before the first storm, whereas the reduction by the storm was four times larger at the 66% emersion time station than at the 80% emersion time station. In contrast to the morphological observations, almost no ecological recovery was observed over the time scale of a month (abundance after October storm was similar to the situation before November storm). Furthermore, the decrease in abundance due to the second storm was smaller compared to the decrease due to the first storm.

## Discussion

Substantial spatiotemporal inhomogeneities in the impact of storm events on the bed level dynamics (bed level changes over $\sim$ days, i.e., variations in the bed level evolution) of intertidal flats have been identified. There is a variation in the spatial extent of storm impacts on intertidal flats, in which variations in storm intensity and duration play a role (similar to Talke and Stacey^27^). We can distinguish the short-term bed level variations around a storm event (the bed level dynamics) and the long-term evolution of an intertidal flat. But also sudden bed level changes during storm events can persist in the long-term evolution, even if they partially recover. Sudden bed level changes during storm events are relevant to consider in the long-term evolution of intertidal flats, as they may compare to years of continuous evolution. Systems that face shortages in sediment supply (such as the Eastern Scheldt) have typically less recovery capacity^29^. Differences in recovery will also relate to differences in tidal forcing (the sole forcing present during calm conditions), which varies over elevations, within an estuary, and between different estuaries (e.g., Fig. 6). Whether or not the impact is in line with the long-term evolution direction (i.e., towards a possible equilibrium) may also affect the recovery. But even in case of a complete morphological recovery, consequences of events can persist in the long term. For example, benthic macrofauna eroded or washed out during events are not necessarily recovered within the recovery time scale of the morphology (Fig. 8). Consequentially, changes in erodibility of the bed can follow^39^.

Consistently across the intertidal flats in the Western Scheldt, the highest bed level variations (i.e., bed level dynamics) occurred generally at the lower parts of the flats (Fig. 3a–g). A similar trend was observed in other studies in this estuary^18,19,21^. In contrast to our decades of measurements with an average measurement interval of 40 days, these studies measured the dynamics with an interval of about one day over approximately one year. The relation between bed level dynamics and the bed elevation persists hence over various measurement intervals (tides—weeks) and is present over various time frames (years—decades). For the Galgeplaat intertidal flat (Eastern Scheldt), such a trend was not observed (Fig. 3h).

To explain differences in bed level dynamics across intertidal flats, we consider the sediment conservation equation for an alongshore uniform intertidal flat (i.e., no gradients in sediment transport in alongshore direction):

1 $$\begin{aligned} {{\partial z_b} \over {\partial t}} = - {1 \over {1 - p}}{{\partial S} \over {\partial x}}, \end{aligned}$$

where $z_b$ is the bed level, *t* time, *p* the porosity, *S* the depth-integrated sediment transport rate, and *x* the distance in cross-shore direction. This equation shows that bed level changes/dynamics are a consequence of gradients in the sediment transport rates. We showed that the forcing processes, and hence also the resulting sediment transport rates, are partly a function of the bed elevation (Figs. 6, 7h). Therefore, it is meaningful to consider the gradients in sediment transport over the bed elevations. Substituting the bed slope ($\beta = {{\partial z_b} /{\partial x}}$) in Eq. (1) results in:

2 $$\begin{aligned} {{\partial z_b} \over {\partial t}} = - {\beta \over {1 - p}}{{\partial S} \over {\partial z_b}}. \end{aligned}$$

This equation expresses the bed level dynamics as the product of (1) the local steepness of an intertidal flat and (2) the gradient in depth-integrated sediment transport (i.e., forcing processes) over the bed elevation. Even though the bed slope may change by morphological changes, it can be considered relatively constant (i.e., as a forcing parameter in Eq. 2) over the time scale of an individual storm event (changes in bed slope occur generally over longer time scales^13^, e.g., Fig. 1b, c). We now consider both components.

Component (1) of Eq. (2) indicates that the bed level dynamics of intertidal flats are linearly proportional to their local bed slope (i.e., a larger bed level variability for a steeper bed). This effect was not considered in recent studies on bed level dynamics patterns on intertidal flats^18–21^. In the Western Scheldt, the bed slope typically decreases by one order of magnitude for elevations from MLW to MHW^6^. This implies that the bed level dynamics decrease by one order of magnitude across these elevations due to variations in bed slope alone, which is precisely the trend observed in Fig. 3. The bed slope gradients are less across the intertidal flats in the Eastern Scheldt^6^, which is (part of) the explanation for the smaller variation in bed level dynamics (i.e., more similar bed level variability) over the vertical in the Eastern Scheldt (Fig. 3). Bed slopes of intertidal flats can evolve significantly over decades^6^. The proportionality between the bed slope and the bed level dynamics implies then also structural changes in bed level dynamics in the long term.

Following component (2) of Eq. (2), the bed level dynamics are also proportional to the variations in the sediment transport (i.e., forcing processes) over bed elevations. The erosion at the lowest elevated frame was over the storm event two orders of magnitudes larger than at the highest elevated frame. The large bed level changes at the lowest elevated frame related to a concurrence of: an almost maximum water depth for which the acting waves still broke, flow velocities that were relatively large for these water depths, higher suspended sediment concentrations than during calm conditions for these water depths, and this all for a large duration (hours). This concurrence of processes, in contrast with the emerged higher elevated frame, was conducive to the large bed level change. Differences in hydrodynamic forcing with the channel were observed as well. As the peak of the storm coincided with low water, the flow in the channel (only 200 m from the MLW line) was almost stagnant (Fig. 4h), whereas on the intertidal flat a flow of 0.7 m/s was still observed. Firstly, this implies that the flow on the flat was not tide-driven but largely wind-driven^32^ instead. Without wind, the flow on the flat would have been almost stagnant as well. The magnitude of the flow velocity on the flat at the peak of the storm is in line with the estimate of wind-driven velocities of order 1/40 of the wind speed^28^, with smaller contributions for larger tidal forcing (tidal forcing is limited in these hours around low water) and larger water depths (where inertia is significant). When the flow was wind-dominated (e.g., at the peak of the storm), the flow was aligned with the wind direction while geometrical constraints by the bathymetry still had to be followed^28,32^ (i.e., predominantly alongshore directed flow). Secondly, the limited flow and wave effects in the channel imply large variations in sediment transport between the channel and the intertidal flat (i.e., implying bed level changes).

Figure 7h revealed that it was not a coincidence that the largest bed level dynamics, resulting from the variations in forcing processes, occurred at the lowest elevation of the intertidal flat. This is a consequence of the forcing processes depending on water depth and bed elevation. Wave impacts relate strongly to water depth^31,32,40,41^, with maximum wave-induced shear stresses at water depths (hereafter referred to as the critical water depths) equal to the significant wave height divided by the wave-breaking index^31^. In shallow water, these maximum bed shear stresses are (almost) not a function of the wave period^31^. At the low elevations of the intertidal flat the tidal flow velocities are small (almost stagnant) for the critical water depths (Fig. 7h), but can be amplified by the wind especially for small tidal velocities^28^. At higher elevations the tidal velocities may peak at the critical water depths (as these velocity peaks occur at higher water levels), although the magnitude of these velocities is there less (Figs. 6, 7h). The smaller importance of the local tidal flow for the morphodynamics at low bed elevations follows also from the measured suspended sediment concentrations, as these concentrations related substantially weaker to the tidal flow at ${\hbox {F}}_{\mathrm{L}}$ than at ${\hbox {F}}_{\mathrm{H}}$ (Fig. 5i, j). From Fig. 7h it was derived that the duration of the effective small critical water depths is the longest just above the MLW line. This means that the long-term likelihood of having a storm event occurring with favourable conditions for large bed level dynamics is the largest for the lowest part of the intertidal flat, which is another part of the explanation for the long-term patterns in bed level dynamics observed in Fig. 3. This is an extension to studies that stressed the crucial role of timing and duration of events^25,27,29^, and that observed the largest bed level changes during short fractions of the tidal period^42^.

A secondary effect of the bed slope on the bed level dynamics through component (2) has to be discussed. Even though the forcing processes are largely a function of the bed elevation, the bed slope may still affect the waves and the flow. The gradient in wave height over bed elevations is negatively proportional to the wave-breaking index $\gamma = H/h$ (with *H* the wave height and *h* the water depth which relates to the bed level: ${\partial h}=-{ \partial z_b}$) within the wave-breaking zone:

3 $$\begin{aligned} {{\partial \mathrm{H}} \over {\partial z_b}} = \gamma {{\partial h} \over {\partial z_b}} = - \gamma . \end{aligned}$$

As $\gamma$ decreases with decreasing bed slopes^32,43^, both the wave height and the gradient in wave height over $z_b$ are smaller for smaller bed slopes. Therefore, a decrease in bed slope implies a decrease in bed level dynamics, just as component (1). For systems in which the flow has large alongshore components (like in the Eastern Scheldt and Western Scheldt), no general relation exists between a change in bed slope and a change in flow velocity, as they depend on the geometry of the channel-flat system. The transport gradients may hence in some cases also increase for milder bed slopes. However, due to the wind, the effect of the bed slope on the tidal velocities is irrelevant for at least the lowest part of intertidal flats, as the tidal flow is there limited at water depths for which waves are important. There, the decrease in bed level dynamics for a decrease in bed slope remains a general concept.

The pattern of decreasing tidal velocities for higher bed elevations within intertidal flats (Fig. 6) is present in many other estuaries^13,17^. Furthermore, the relatively long duration of water levels around low water is a general consequence of the shape of a tidal wave. Therefore, in other estuaries similarities in the likelihood distribution of the bed level dynamics across intertidal flats are expectable. Apart from gradients in sediment transport and the bed slope, bed level dynamics are also affected by aspects such as grain size variability^17,44^, benthos^45–47^, and bed erodibility^39^. Bed level dynamics may also follow from sediment transported through lateral circulation^48^, which is especially an important mechanism during fair weather in estuaries with highly turbid channels^49^. Differences in wave climate induce differences in bed level dynamics around an individual intertidal flat, for example, following variations in fetch (which is a function of location and wind direction)^41^. Spatial variations in the importance of wave-current interaction^50,51^ play a role too. For example, waves that reached the high part of the studied intertidal flat faced generally substantially larger tidal currents in the channel/foreshore than waves that reached the low part of the flat, considering similar water depths (Figs. 5h, 7e).

In contrast to previous studies that solely compared the wave and tidal forcing to explain bed level dynamics patterns^18–21^, we showed that it is important to include wind-driven flow when explaining these patterns. For the lowest elevations of an intertidal flat, the wind essentially replaces the small tidal forcing for water depths at which waves are most efficient (Fig. 7h). Including a full wind/wave climate in long-term morphological simulations is computationally very expensive. Instead, given the existence of patterns in the hydrodynamic forcing processes over bed elevation and water depth (Fig. 7h), it is worth to investigate whether these effects could be parametrized in these models (using the local distributions of the forcing processes). Furthermore, for intertidal flats, it was not demonstrated before that patterns in bed level dynamics can be explained partly by the linear proportionality with the bed slope, and not only by the gradients in sediment transport. The identified spatiotemporal inhomogeneities in forcing processes and bed level dynamics can have direct implications for inhomogeneities in benthos (e.g., Fig. 8), which in turn can affect the bed level dynamics^45–47^. Therefore, not only variations in bed level^22,23^, but also variations in storm impacts, drive variations in benthic macrofauna. When the bed level or bed slope of an intertidal flat evolves in the long term, structural changes in bed level dynamics will follow with related effects on benthic macrofauna.

### Study areas

This study focused on various intertidal areas within the Eastern Scheldt and Western Scheldt (both located in the Netherlands; see Fig. 1). Both estuaries are connected to the North Sea. River inflow to the Eastern Scheldt has been blocked by dams. Since 1986, the estuary has a storm surge barrier at its mouth which closes under severe storm conditions. The mean tidal range in the Eastern Scheldt ranges from 2.5 m at its mouth up to 3.5 m in one of its branches. The Western Scheldt has an average river discharge of about $100~{\hbox {m}}^3/\hbox {s}$, which is at the mouth only approximately 0.1% of the tidal discharge. The mean tidal range in the Western Scheldt ranges from 3.5 m at its mouth up to 5 m near the Belgian border (upstream). For the deepening and maintenance of the navigation channel of the Western Scheldt, sediment relocations take place. The human interventions in both systems affect the long-term evolution of the intertidal flats. Although these estuaries are close and similar in size, they develop differently. During the past decades, the intertidal flats in the Western Scheldt mainly raised and steepened, while in the Eastern Scheldt the intertidal flats lowered and flattened out^6,8,52^. As these are two neighbouring estuaries, their wind climates are relatively comparable, with generally the strongest winds from the southwest and wind speeds exceeding 10 m/s roughly 1/8th of the time. Most waves in these systems are locally generated, with a peak period of a few seconds.

## Methods

Rijkswaterstaat (the Dutch Ministry of Infrastructure and the Environment) has been extensively measuring the long-term morphological changes of intertidal flats over various temporal and spatial scales for decades. Annual dGPS-RTK elevation transects ($2\sigma$ of 6 cm)^53^ have been measured on intertidal flats in both the Eastern Scheldt and Western Scheldt since 1987. Additionally, point-elevation measurements have been measured at fixed locations on the intertidal flats of both estuaries since 1984. Time series of the Zuidgors and Galgeplaat intertidal flats are used in this study to analyse the effect of storm events on the long-term evolution of these areas. Furthermore, the data of additional intertidal flats in the Western Scheldt are systematically analysed to study a possible relation between bed elevation and the bed level dynamics (variations in the bed level evolution). The average measurement interval was 40 days. These data have been gathered with Sediment Erosion Bars until 2008, and with RTK-dGPS afterwards. For every measurement, 15 samples were averaged within a 2 m radius, such that local irregularities were excluded and centimetre precision was achieved. The bathymetric maps in this study are based on single beam and LiDAR measurements ($2\sigma$ of 50 cm and 30 cm, respectively)^53,54^.

Complementary to these long-term morphological datasets, we have deployed instruments to measure the hydro-morphodynamics over 1 month (15 November–15 December 2016). These data are used to study the hydro-morphodynamic processes during an individual storm event, and the calm period surrounding this event. The instruments were placed along a cross-shore transect on the Zuidgors intertidal flat in the Western Scheldt (Figs. 1a, 4a). Two measurement frames were deployed on the intertidal flat (${\hbox {F}}_{\mathrm{H}}$ 1.0 m above MSL; ${\hbox {F}}_{\mathrm{L}}$ 1.6 m below MSL). In the adjacent channel, an Acoustic Doppler Current Profiler (ADCP) was deployed (6.8 m below MSL), which measured velocities on a 10 min interval with a 0.5 m bin size.

Each frame contained an Acoustic Doppler Velocimeter that sampled bed elevations at 10 min intervals. Each frame also contained a vertical array of two (${\hbox {F}}_{\mathrm{H}}$) or four (${\hbox {F}}_{\mathrm{L}}$) Optical Backscatter Sensors (OBSes) that were at least 50 cm spaced. After a post-campaign calibration against water samples with suspended sediment from the site, the OBSes provided time series of suspended sediment concentrations. Near each frame, an upward-looking ADCP was deployed which measured the velocities in the water column on a 10 min interval with a 0.1 m bin size. Also a 10 Hz pressure sensor was deployed in the bed near each frame to measure water levels and waves (these were deployed for a longer period, October 2016–February 2018). The pressure signals were corrected for measured atmospheric pressure fluctuations. Wave-induced shear stresses were computed following Soulsby^55^. Water levels measured by Rijkswaterstaat (retrieved from waterinfo.rws.nl) at the nearby tidal gauge station Borssele (7 km downstream of Zuidgors) were used for the analyses that required continuous long-term water level data as the instruments on the intertidal flat emerged. Wind data was retrieved from the KNMI Vlissingen wind station (17 km west of Zuidgors).

For the derivation of system-wide peak velocities on the intertidal flats of both estuaries (Fig. 6), depth-averaged numerical simulations with the Delft3D model^56^ are used. While these simulations are the same as presented in De Vet et al.^6^, we consider in this study the peak velocities in relation to the bed level, instead of visually on a map. For both estuaries, the simulations had been calibrated and validated with measured hydrodynamics^57,58^.

The benthic macrofauna (i.e., macroinvertebrates $> 1~ \hbox {mm}$) was sampled (3 sediment core replicates, Ø10 cm, 30 cm depth) on the Zuidgors intertidal flat before and after two storms in 2017 (October and November). Samples were taken over a cross-shore transect 1 km west of and parallel to the 2016 measurement frames. The benthic macrofauna samples were determined to species level and the abundance ($\hbox {N}/{\hbox {m}}^2$) of the community was calculated. The six sampling stations were equally distributed in emersion time (between 10 and 80% emersion time). The community was characterized by high abundances of short-living species, like the mudshrimp *Corophium* sp. ($< 1$ year life span) and the red thread worm *Heteromastus filiformis* (1–2 year life span).
